# Diosmetin Modulates EMT-Associated Plasticity and Fibroblast-Activation Markers in Parallel Breast Cancer In Vitro Models

**DOI:** 10.3390/molecules31122111

**Published:** 2026-06-16

**Authors:** Monika Michalczyk, Joanna Kubik, Aleksandra Józefczyk, Magdalena Iwan, Ewelina Humeniuk, Grzegorz Adamczuk, Mariola Michalczuk, Barbara Madej-Czerwonka, Maciej Czerwonka, Agnieszka Korga-Plewko

**Affiliations:** 1 Doctoral School, Medical University of Lublin, 7 Chodzki Street, 20-093 Lublin, Poland; 51802@student.umlub.pl; 2 Independent Medical Biology Unit, Faculty of Pharmacy, Medical University of Lublin, 8b Jaczewskiego Street, 20-090 Lublin, Poland; joanna.kubik@umlub.edu.pl (J.K.); ewelina.humeniuk@umlub.edu.pl (E.H.); mariola.michalczuk@umlub.edu.pl (M.M.); 3 Department of Pharmacognosy with Medicinal Plants Garden, Faculty of Pharmacy, Medical University of Lublin, 1 Chodzki Street, 20-093 Lublin, Poland; ajozefczyk@pharmacognosy.org; 4 Department of Toxicology, Faculty of Pharmacy, Medical University of Lublin, 6 Chodzki Street, 20-093 Lublin, Poland; magdalena.iwan@umlub.edu.pl; 5 Department of Physiology and Toxicology, Institute of Medical Science, Faculty of Medicine, John Paul II Catholic University of Lublin, 1H Konstantynow, 20-708 Lublin, Poland; grzegorz.adamczuk@kul.pl; 6 Human Anatomy Department, Faculty of Medicine, Medical University of Lublin, 4 Jaczewskiego Street, 20-090 Lublin, Poland; barbara.madej-czerwonka@umlub.edu.pl; 7 Department of General, Oncological, Gastroenterological and Transplant Surgery, University Hospital in Krakow, Jagiellonian University, 2 Jakubowski Street, 30-688 Krakow, Poland; mc.red@interia.pl

**Keywords:** anticancer activities, breast cancer, cancer-associated fibroblasts, diosmetin, epithelial–mesenchymal transition, natural product, senescence

## Abstract

Metastasis remains the leading cause of mortality in breast cancer and is closely linked to epithelial–mesenchymal transition (EMT) and tumor microenvironment (TME)-associated processes. Diosmetin (DT), the active metabolite of diosmin, a widely used venoactive drug, has emerged as a potential anticancer agent. Building on our previous findings demonstrating that DT enhances doxorubicin efficacy, this study investigated its effects on tumor cell plasticity and stromal activation-associated responses. EMT was induced in MCF-7 cells, while a stromal model was established by TGF-β-mediated activation of BJ fibroblasts toward a cancer-associated fibroblast (CAF)-like phenotype. Additionally, doxorubicin-induced senescence was generated in fibroblasts. Migration assays and quantitative real-time PCR were used to assess functional and transcriptional changes. EMT induction resulted in decreased *CDH1* expression and increased levels of *VIM*, *MMP2*, *MMP9*, *IL-6*, and *HIF-1A*, accompanied by enhanced migratory activity. DT attenuated TGF-β-induced CAF-like activation, as reflected by reduced expression of *ACTA2*, *HGF*, *MMP2*, *MMP9*, and *IL6*, and modulated hyaluronan turnover-related genes. Moreover, DT partially alleviated selected senescence-associated features in doxorubicin-treated fibroblasts. Collectively, these findings indicate that DT modulates EMT-associated plasticity and stromal activation-related responses in parallel in vitro models. Given its origin as a metabolite of a clinically used compound and its previously demonstrated chemosensitizing properties, DT may warrant further investigation as a potential adjunctive agent to modulate tumor- and stromal-associated processes in breast cancer.

## 1. Introduction

Diosmin is a semi-synthetic flavonoid widely used in clinical practice as a venoactive agent to treat chronic venous insufficiency, hemorrhoidal disease, and microcirculatory disorders [1]. Owing to its long-standing clinical use and well-established safety profile, diosmin is an attractive candidate for drug repurposing strategies [2,3]. After oral administration, diosmin is metabolized by the intestinal microbiota into diosmetin (DT) [4], its biologically active aglycone. Diosmetin (3′,5,7-trihydroxy-4′-methoxyflavone) is an O-methylated flavone whose biological activity is linked to the presence and position of hydroxyl and methoxy groups within the flavonoid scaffold, which may influence antioxidant capacity, signaling modulation, and metabolic stability [5,6]. Growing interest in drug repurposing—the identification of new therapeutic indications for approved or clinically used compounds—has prompted investigations into the potential anticancer properties of DT.

Accumulating evidence indicates that DT exerts anti-inflammatory, antioxidant, and antiproliferative effects in various cancer models. It has been shown to induce apoptosis, modulate the PI3K/Akt and MAPK signaling pathways, and suppress tumor cell growth in several malignancies [7,8,9,10,11,12]. In our previous study, we demonstrated that DT enhances the efficacy of doxorubicin (DOX) in MCF-7 breast cancer cells by promoting DNA damage accumulation and inhibiting P-glycoprotein activity, a key mediator of multidrug resistance [13]. These findings support the notion that DT may act as a chemosensitizing agent, thereby potentiating conventional chemotherapy. However, whether DT influences tumor cell plasticity and associated stromal response remains largely unexplored.

Breast cancer progression and metastatic dissemination are driven not only by intrinsic genetic alterations but also by dynamic phenotypic reprogramming and mutual interactions within the tumor microenvironment (TME) [14,15,16]. A central mechanism underlying tumor invasiveness is epithelial–mesenchymal transition (EMT), a process characterized by the loss of epithelial markers such as E-cadherin (CDH1), the acquisition of mesenchymal traits, including VIM expression, and the activation of matrix metalloproteinases (MMPs) [17,18,19]. EMT enhances migratory capacity, invasiveness, and resistance to therapy. Moreover, EMT is closely associated with inflammatory signaling and hypoxia-responsive pathways, including activation of hypoxia-inducible factor-1 alpha (HIF-1α), which collectively promote tumor aggressiveness [20].

Importantly, EMT is not solely a tumor cell-autonomous program but is strongly influenced by the surrounding stroma [21,22]. Cancer-associated fibroblasts (CAFs) identified by markers such as alpha-smooth muscle actin (α-SMA), encoded by the *ACTA2* gene, represent a major stromal component of the TME and contribute to tumor progression through the secretion of pro-inflammatory cytokines (e.g., IL-6), growth factors such as hepatocyte growth factor (HGF), and extracellular matrix-remodeling enzymes [23,24,25]. Moreover, CAFs interact with receptors on cancer cells, triggering a signaling cascade that ultimately enhances cancer cell survival, invasion, proliferation, and resistance to chemotherapy [26]. In parallel, dysregulated hyaluronan (HA) metabolism, controlled by hyaluronan synthases (HAS) and hyaluronidases (HYAL), facilitates extracellular matrix remodeling and supports tumor cell motility [27,28,29]. Furthermore, therapy-induced cellular senescence and the associated senescence-associated secretory phenotype (SASP) generate a pro-inflammatory environment that can reinforce EMT, promote CAF activation, and enhance metastatic potential [30,31].

DT has been reported to exert cytotoxic and chemosensitizing effects; however, its impact on EMT-associated plasticity, stromal activation, extracellular matrix remodeling, and SASP-related signaling in breast cancer remains insufficiently characterized. Given the established clinical availability of diosmin and the demonstrated ability of DT to enhance chemotherapy efficacy, elucidating additional mechanisms by which DT modulates tumor and stromal compartments may significantly strengthen its translational relevance as a repurposed adjunct in breast cancer therapy.

Therefore, the present study aimed to investigate whether DT modulates EMT in MCF-7 breast cancer cells and influences fibroblast activation toward a CAF-like phenotype in an in vitro model. We examined transcriptional changes in EMT markers, inflammatory mediators, hypoxia-related factors, and HA metabolism genes, alongside functional migration assays. Additionally, we evaluated the effect of DT on DOX-induced senescence and SASP-associated features. While previous studies on diosmetin have primarily focused on its cytotoxic, antioxidant, and chemosensitizing properties, its potential role in EMT-associated plasticity, fibroblast activation, extracellular matrix remodeling, and senescence-related stromal responses remains insufficiently characterized. To our knowledge, this is the first study to functionally evaluate these tumor- and stromal-associated processes in parallel in vitro breast cancer models in the context of diosmetin treatment. Accordingly, the present work extends the current understanding of diosmetin beyond its previously described cytotoxic and chemosensitizing effects.

## 2. Results

### 2.1. Validation of EMT-Associated Phenotype

**Figure 1:**
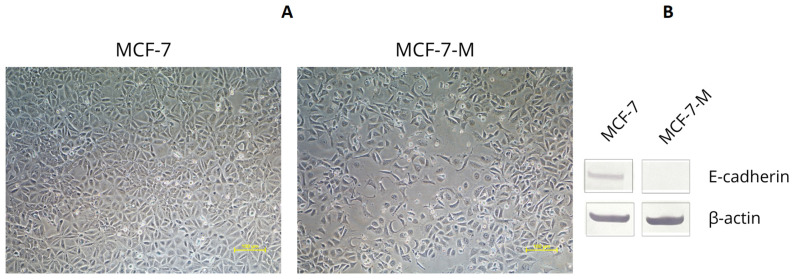
Validation of the EMT-associated model in MCF-7 cells. (**A**) Representative phase-contrast microscopy images of control MCF-7 cells and EMT-induced MCF-7 cells showing morphological changes associated with EMT, including reduced epithelial organization and increased spindle-like morphology. Scale bars = 100 µm. (**B**) Western blot analysis of E-cadherin protein expression in control and EMT-induced MCF-7 cells. β-actin was used as a loading control.

Treatment with the EMT-inducing medium resulted in visible morphological changes in MCF-7 cells, including loss of cobblestone-like epithelial morphology, loosening of intercellular contacts, and acquisition of a more elongated cell shape consistent with EMT-associated phenotypic plasticity (Figure 1A). These phenotypic changes were accompanied by reduced E-cadherin protein expression, confirming induction of an EMT-associated phenotype (Figure 1B and Figure S1).

### 2.2. Effects of EMTi and DT40 on the Expression of EMT Markers in the MCF-7 Line in Quantitative Real-Time PCR Analysis (qRT-PCR)

Treatment with the EMT inducer (EMTi) resulted in a marked shift in gene expression in MCF-7 cells, consistent with EMT features. Compared with control cultures, MCF-7-M cells exhibited a clear reduction in *CDH1* (ECAD) expression to approximately 50% of baseline, accompanied by a strong increase in VIM expression (~8–9-fold), indicating loss of epithelial characteristics and acquisition of a mesenchymal-like phenotype.

In parallel, EMT induction led to upregulation of extracellular-matrix-remodeling genes. Both *MMP2* and *MMP9* were markedly increased (nearly 3-fold). Additionally, *IL-6* expression was substantially elevated (~2-fold), supporting the activation of a pro-inflammatory program. *HIF1A* expression was also increased, although to a more moderate extent compared to other EMT markers.

DT treatment alone (MCF-7+DT40) was associated with heterogeneous transcriptional responses under basal conditions. Instead, it induced gene-specific effects: *CDH1, VIM*, *MMP2*, *HAS*, and *HIF1A* showed moderate increases compared to control cells, whereas *MMP9*, *IL-6*, and *HYAL* were reduced. These findings indicate that DT treatment alone induced gene-specific transcriptional alterations in MCF-7 cells under basal conditions.

In EMT-induced cultures (MCF-7-M+DT40), DT partially reversed the EMT-associated expression pattern. *CDH1* expression was increased to levels exceeding those observed in control cells (~1.5-fold), while *VIM* expression was markedly reduced compared with MCF-7-M cells, although it remained elevated relative to untreated controls (~2.75-fold). Similarly, *MMP2*, *MMP9*, and *IL-6* levels were significantly decreased in the co-treatment cultures, indicating attenuation of ECM remodeling and inflammatory signaling.

A comparable trend was observed for *HIF1A*, whose expression was reduced in MCF-7-M+DT40 cells relative to EMT-induced cultures and approached baseline levels.

**Figure 2:**
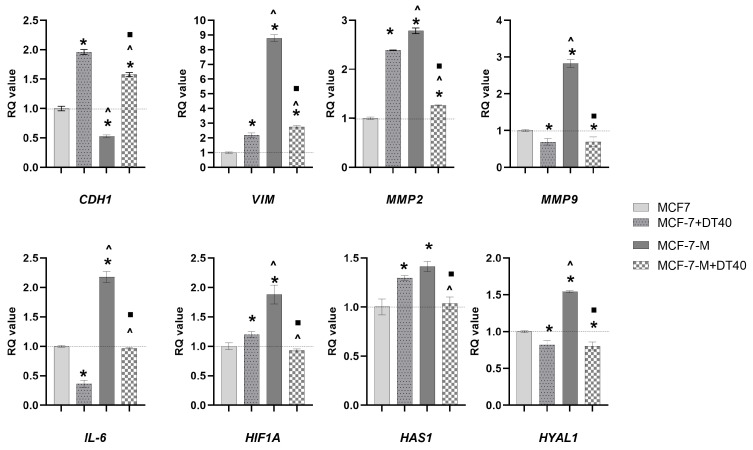
Relative mRNA expression levels of selected genes in the MCF-7 cell lines. The results are presented as relative quantification (RQ) values and expressed as mean ± SD. *B-ACT* and *18SN5* were used as reference genes. Cells were treated for 48 h with EMTi (epithelial–mesenchymal transition inductor), 40 µM DT, or a combination of EMTi and DT40. To compare more than two groups, one-way analysis of variance (ANOVA) and post hoc multiple comparisons based on Tukey’s HSD test were used. * *p* < 0.05 vs. MCF-7; ^ *p* < 0.05 vs. MCF-7-DT40; ^■^ *p* < 0.05 vs. MCF-7-M vs. MCF-7-M+DT40. MCF-7 represents the parental human breast adenocarcinoma cell line. MCF-7-M denotes EMT-induced MCF-7 cells, MCF-7+DT40 indicates parental cells treated with DT40, whereas MCF-7-M+DT40 refers to EMT-induced MCF-7 cells treated with DT40.

Genes involved in HA metabolism displayed differential regulation. EMT induction increased both *HAS* and *HYAL* expression, whereas DT co-treatment reduced their levels near the control baseline (Figure 2, Table S1).

### 2.3. Effects of EMT Induction and Diosmetin on MCF-7 Cell Migration Assessed by Scratch-Wound Assay

**Figure 3:**
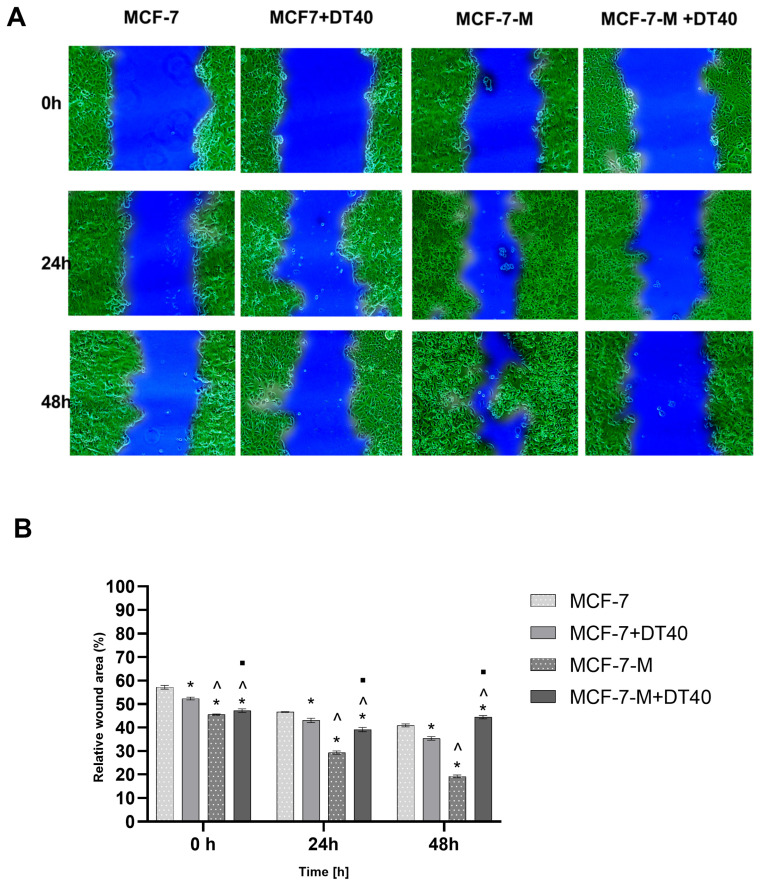
Cell migration of MCF-7 and MCF-7-M cells in the scratch-wound assay following DT40 treatment. Cells were incubated with DT40 for 48 h. (**A**) Representative microscopic images acquired at ×40 magnification at 0 h, 24 h, and 48 h, illustrating relative wound area over time. The green area corresponds to cells, whereas the blue area represents the wound region. Image analysis was performed using NIS Elements software version 3.22. (**B**) Quantitative analysis of relative wound area (%) over time. Lower values indicate increased cell migration and wound closure. Data are presented as mean ± SD. To compare more than two groups, two-way analysis of variance (ANOVA) and post hoc multiple comparisons based on Tukey’s HSD test were used. * *p* < 0.05 vs. MCF-7; ^ *p* < 0.05 vs. MCF-7-DT40; ^■^ *p* < 0.05 vs. MCF-7-M vs. MCF-7-M+DT40. MCF-7 represents the parental human breast adenocarcinoma cell line. MCF-7-M denotes EMT-induced MCF-7 cells, MCF-7+DT40 indicates parental cells treated with DT40, whereas MCF-7-M+DT40 refers to EMT-induced MCF-7 cells treated with DT40.

A scratch-wound assay was performed to assess the migratory capacity of MCF-7 cells under different experimental conditions. Representative micrographs at 0, 24, and 48 h illustrate changes in wound area over time (Figure 3A,B, Table S2). At 0 h, all cultures displayed a clearly defined wound area of comparable width, confirming consistent scratch formation.

In control cultures, a gradual wound decrease in relative wound area was observed over time, reflecting baseline migratory activity. A similar pattern was observed in DT-treated cultures (MCF-7+DT40), where the reduction in wound area did not markedly differ from that of control cells, suggesting a limited effect of DT on basal migration under these conditions.

In contrast, MCF-7-M cultures exhibited a more pronounced decrease in wound area, particularly at 48 h, indicating increased migratory capacity associated with EMT induction.

In EMT-induced cultures treated with DT (MCF-7-M+DT40), the reduction in wound area was less pronounced compared to MCF-7-M cultures. The wound region remained larger at corresponding time points, indicating a partial attenuation of EMT-associated migratory behavior.

Overall, these observations indicate that DT had minimal effects on the basal migratory behavior of MCF-7 cells but was associated with attenuation of the EMT-induced increase in migration.

### 2.4. Diosmetin Effect on Cell Dynamics in Real-Time Impedance Assay

**Figure 4:**
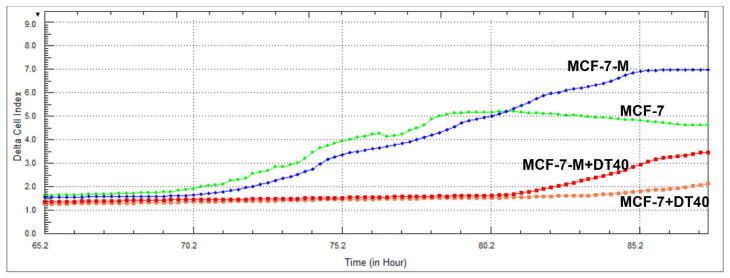
Real-time impedance-based analysis of cellular responses to diosmetin (DT) and the EMTi using the xCELLigence RTCA system. Impedance values are presented as Δ Cell Index (CI), which reflects dynamic changes in cell migration. Cells were treated for 96 h with EMTi and 40 μM of DT or a combination (EMTi + DT). MCF-7 represents the parental human breast adenocarcinoma cell line. MCF-7-M denotes EMT-induced MCF-7 cells, MCF-7+DT40 indicates parental cells treated with DT40, whereas MCF-7-M+DT40 refers to EMT-induced MCF-7 cells treated with DT40.

To functionally evaluate the migratory dynamics of MCF-7 cells, real-time impedance measurements were performed using the RTCA system. The migration-driven changes were analyzed using the Delta Cell Index (ΔCI), thereby normalizing migration profiles across experimental conditions. As shown in the impedance-based migration curves (Figure 3), induction of a mesenchymal-like phenotype in the MCF-7-M culture resulted in a rapid and sustained increase in ΔCI values, indicating enhanced migratory activity compared with control cultures. In contrast, the untreated MCF-7 control culture followed a standard, baseline migration pattern. The impact of DT on motility was clearly observed in the MCF-7+DT40 group, which exhibited a significantly flattened ΔCI curve, indicating that the compound moderately reduced the cells’ migratory capacity. Most importantly, in the MCF-7-M+DT40 group, DT partially attenuated the migration-enhancing effects of the EMT. The CI trajectory for this combined treatment remained substantially lower than that of the MCF-7-M group, demonstrating that DT significantly attenuates transition-driven migration. These real-time data provide functional evidence that DT attenuates cellular motility. By effectively reducing Cell Index values relative to the MCF-7-M culture, DT suppressed the functional migration program triggered during EMT (Figure 4).

### 2.5. Assessment of Apoptosis/Cell Viability Following DT Treatment

To exclude overt cytotoxicity as a major contributor to the observed EMT-associated effects, apoptosis analysis was performed in MCF-7 cells treated with 40 µM DT under the same experimental conditions used throughout the study. No marked increase in apoptotic cell death was observed compared with control cultures (Figure S2), supporting the interpretation that the observed transcriptional and functional changes reflect phenotypic modulation rather than extensive cytotoxicity.

### 2.6. Effects of DT on the Expression of CAFs-Related Markers in the BJ Cell Line in Quantitative Real-Time PCR Analysis (qRT-PCR)

Fibroblasts exposed to TGF-β stimulation (CAFs-like BJ) demonstrated a marked activation profile, although the extent and direction of changes varied across markers. The expression of *ACTA2* increased approximately ~1.5-fold compared to control BJ cells, indicating a shift towards a myofibroblast-like phenotype. Similarly, *HGF* level was elevated (~4-fold), confirming fibroblast activation. Matrix-remodeling enzymes were also induced. *MMP2* expression increased to ~4-fold, while *MMP9* showed the strongest induction (~8-fold), suggesting enhanced breakdown of the extracellular matrix. Concurrently, *IL-6* expression was markedly elevated (~7-fold), consistent with a pro-inflammatory phenotype. Treatment with DT (CAFs-like BJ+DT40) partially reduced this activation. The expression levels of *ACTA2*, *HGF*, and *HIF1A* decreased below the control level, whereas *MMP2*, *MMP9*, and *IL-6* decreased compared to the CAFs-like BJ culture; however, they remained above the baseline levels observed in control BJ cells. Notably, the reduction in *ACTA2* suggests a partial inhibition of myofibroblast differentiation. Genes involved in HA metabolism showed a distinct pattern. In the CAFs-like BJ cultures, *HAS* expression was significantly reduced compared to the control, while HYAL was clearly upregulated (~1.5-fold), indicating increased HA degradation. Following DT treatment, *HYAL* expression decreased (~60% below the control level), whereas HAS expression remained low.

**Figure 5:**
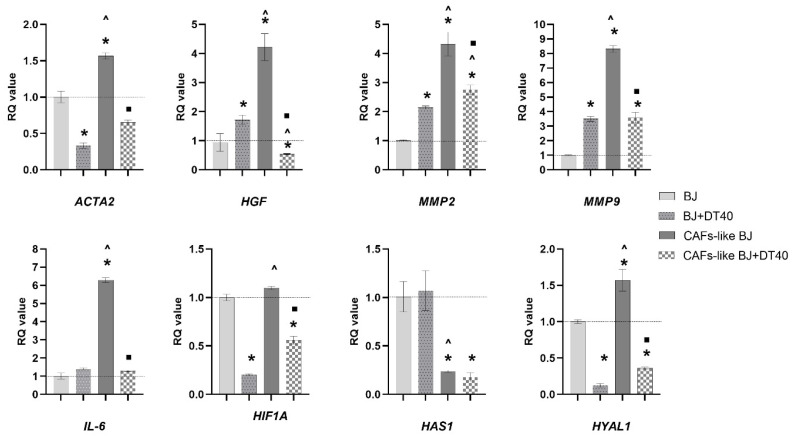
Relative mRNA expression level of selected genes in the BJ cell line. BACT and RNA18SN5 were used as reference genes. Cells were treated for 48 h with 10 ng/mL TGF-β, 40 µM DT, or a combination of TGF-β and DT40. The results are presented as relative quantification (RQ) values and expressed as mean ± SD. To compare more than two groups, two-way analysis of variance (ANOVA) and post hoc multiple comparisons based on Tukey’s HSD test were used. * *p* < 0.05 vs. BJ; ^ *p* < 0.05 vs. BJ+DT40; ^■^ *p* < 0.05 vs. CAFs-like BJ vs. CAFs-like BJ+DT40. BJ represents the parental human skin fibroblast cell line. CAFs-like BJ denotes the cancer-associated fibroblast-like variant of BJ cells, characterized by a TGF-β-activated phenotype. BJ+DT40 indicates parental cells treated with 40 µM of DT, whereas CAFs-like BJ+DT40 refers to activated (CAFs-like) variant treated with DT40.

Importantly, DT alone (BJ+DT40) did not uniformly suppress all analyzed markers. A modest increase in *MMP2*, *MMP9*, and *HGF* expression was observed compared with control BJ cells. Conversely, *ACTA2*, *HIF1A*, and *HYAL* expression levels were significantly reduced in DT-treated fibroblasts. These findings indicate that DT treatment was associated with heterogeneous, gene-specific transcriptional responses under basal conditions. Notably, despite the observed increases in selected genes under unstimulated conditions, DT attenuated TGF-β-induced upregulation of activation-associated markers in stimulated fibroblasts. (Figure 5, Table S3).

### 2.7. SA-β-Gal Staining for Senescence Detection in Normal BJ Fibroblasts

**Figure 6:**
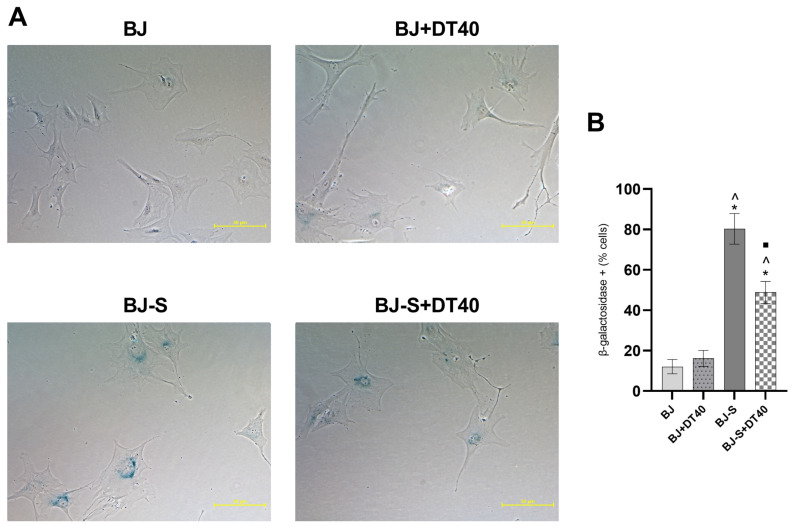
(**A**) Representative microscopic images of BJ fibroblasts stained for senescence-associated β-galactosidase (SA-β-Gal) under different treatment conditions. (**B**) Quantification of SA-β-Gal-positive cells (% of cells), expressed as mean ± SD from three independent experiments. Senescent cells (BJ-S) were compared with non-senescent BJ cells, as well as their respective DT40-treated groups. Statistical analysis was performed using one-way ANOVA followed by Tukey’s post hoc test. * *p* < 0.05 vs. control (BJ); ^ *p* < 0.05 vs. BJ+DT40; ^■^ *p* < 0.05 vs. BJ-S. The experimental groups were defined as follows: BJ—untreated cells (control); BJ+DT40—fibroblasts treated with 40 μM diosmetin; BJ-S—senescent fibroblasts; BJ-S+DT40—senescent fibroblasts treated with 40 μM DT40.

Senescence-associated β-galactosidase (SA-β-gal) staining demonstrated a significant increase in the proportion of senescent cells in BJ-S cultures compared to control BJ fibroblasts (Figure 6A, Table S4). This was accompanied by characteristic morphological changes, including enlarged and flattened cell shape.

DT treatment alone (BJ+DT40) did not significantly affect the percentage of SA-β-gal-positive cells relative to untreated BJ controls (16.2% vs. 12.07%), indicating no induction of senescence under basal conditions. In cells incubated with DOX to induce senescence, 80.3% of cells were positive (Figure 6A).

In contrast, treatment of senescent fibroblasts with DT (BJ-S+DT40) resulted in a significant reduction in the proportion of SA-β-gal-positive cells compared to BJ-S cultures. Although the percentage of senescent cells remained elevated relative to BJ controls (48.83% vs. 12.07%), a clear decrease was observed following DT treatment (Figure 5).

These findings indicate that DT does not promote senescence in resting fibroblasts but partially reduces the senescent phenotype in BJ-S cells.

### 2.8. Effect of DT on the Proliferation of Senescent BJ Fibroblasts

**Figure 7:**
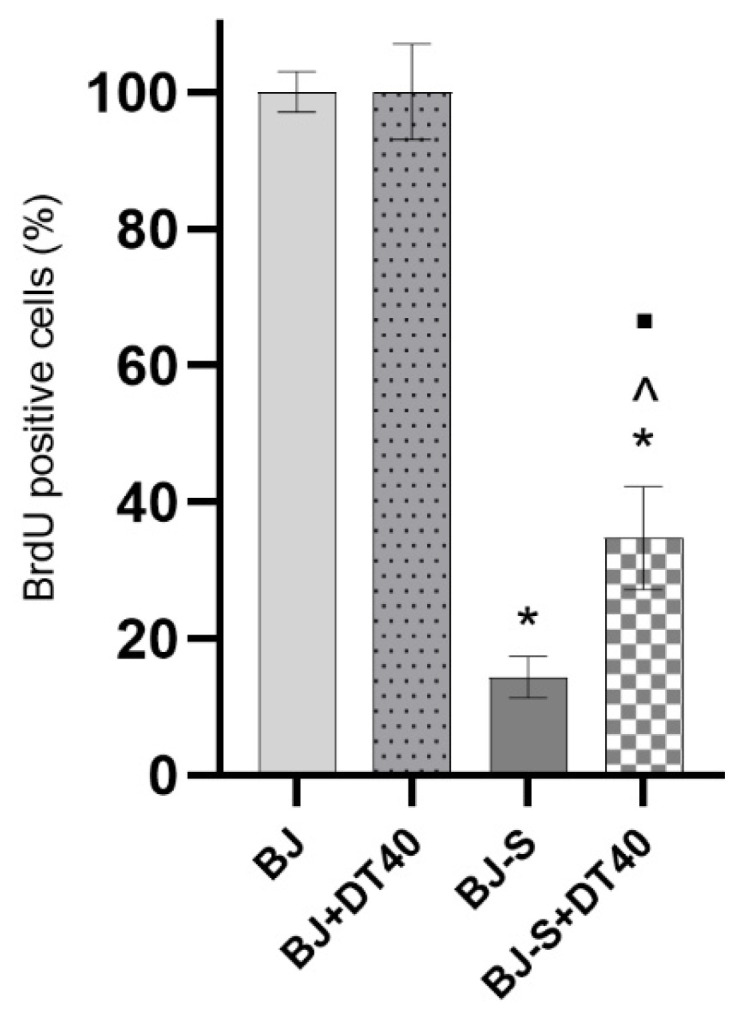
Evaluation of proliferation in BJ fibroblasts using BrdU incorporation assay. Cell proliferation was assessed by BrdU incorporation and expressed as the percentage of BrdU-positive cells. BJ fibroblasts were treated for 48 h with 1 µM doxorubicin (DOX) and/or 40 µM diosmetin (DT40). Data are presented as mean ± SD from three independent experiments. Statistical analysis was performed using one-way ANOVA followed by Tukey’s post hoc test. * *p* < 0.05 vs. BJ; ^ *p* < 0.05 vs. BJ+DT40; ^■^ *p* < 0.05 vs. BJ-S. The experimental groups were defined as follows: BJ—untreated cells (control); BJ+DT40—cells treated with 40 µM DT40; BJ-S—cells treated with 1 µM DOX (senescent BJ cells); BJ-S+DT40—DOX-induced senescent BJ cells treated with 40 µM DT40.

Cell proliferation was assessed using the BrdU incorporation assay. As shown in Figure 7, control BJ fibroblasts and cells treated with DT40 alone exhibited high levels of proliferation, with BrdU-positive cells reaching approximately 97–100%, indicating that DT40 did not significantly affect proliferative capacity under these conditions. In contrast, treatment with DOX (BJ-S) resulted in a marked decrease in BrdU incorporation to approximately 15%, confirming effective induction of senescence and proliferation arrest. Importantly, treatment of senescent cells with DT40 (BJ-S+DT40) significantly increased the percentage of BrdU-positive cells to approximately 30–40%, compared to BJ-S cells. However, this level remained significantly lower than in control and BJ+DT40 groups (^ *p* < 0.05), indicating only partial restoration of proliferative activity (Figure 7, Table S5).

## 3. Discussion

Breast cancer metastasis is a complex, multistep process driven not only by intrinsic tumor cell plasticity but also by dynamic interactions within the TME. In this study, we demonstrate that DT exerts multi-level regulatory effects by simultaneously modulating EMT-associated tumor cell behavior and stromal activation.

DT is a natural polyphenol with potent antioxidant and anti-inflammatory properties [32,33,34]. Given its pharmacological effects and safety, DT has been widely used as a dietary supplement and medication, primarily for vascular disorders that often accompany cancer and oncological treatment (1). Previous studies have reported the effectiveness of DT in cancer treatment [7,8,9,10,35,36]. In our prior work, we found that DT potentiates DOX activity in MCF-7 breast cancer cells by promoting DNA damage accumulation and inhibiting P-glycoprotein, a key driver of multidrug resistance [13]. However, its role in inducing EMT in breast cancer cells remains underexplored. Therefore, in the present study, we examined the effects of DT on cell proliferation, migration, EMT in cancer cells, and the activation of fibroblasts toward a CAF-like phenotype and senescence.

The 40 µM diosmetin concentration used in this study was selected based on our previous dose–response and apoptosis profiling in MCF-7 cells, where it fell within a sub-lethal modulatory window below the apoptotic threshold. Although this concentration exceeds typical levels of free circulating diosmetin in peripheral plasma, its local relevance within tumor tissue may be partially supported by tumor-associated metabolic activation. Following oral administration, diosmetin predominantly circulates as glucuronide conjugates [37], whereas elevated extracellular β-glucuronidase activity within tumor and stromal compartments may locally regenerate the active aglycone form [38]. Nevertheless, the applied concentration should be interpreted primarily within the context of exploratory in vitro modeling rather than as a direct representation of systemic plasma exposure levels. Combined transcriptional analysis, morphological alterations, migration-based functional assays, and partial protein-level validation via E-cadherin assessment supported the observed EMT phenotype. EMT induction resulted in a transcriptional shift consistent with increased cellular plasticity, including decreased *CDH1* expression and upregulation of mesenchymal, inflammatory, and extracellular matrix-remodeling markers, such as *VIM*, *MMP2*, *MMP9*, and *IL-6*. These molecular changes were accompanied by enhanced migratory capacity, confirming the acquisition of a migratory and mesenchymal-like phenotype. Importantly, DT treatment significantly attenuated these effects at both the transcriptional and functional levels, suggesting its role as a modulator of EMT-associated tumor cell behavior.

DT treatment was associated with partial restoration of CDH1 expression and reduced expression of VIM, MMP2, and MMP9. These changes suggest attenuation of selected EMT-associated molecular features. However, the observed responses did not fully restore the transcriptional profile of control MCF-7 cells, indicating only partial reversal of EMT-associated alterations. The downregulation of MMP2 and MMP9 is particularly relevant because these proteases directly degrade the extracellular matrix and facilitate tumor cell invasion [39,40]. Similarly, increased vimentin expression has been strongly associated with enhanced motility and poor clinical outcomes in breast cancer [41].

Taken together, these results suggest that DT predominantly modulates selected functional and molecular features associated with EMT rather than fully reversing EMT-associated alterations, which is consistent with the current understanding of EMT as a dynamic spectrum of cellular states [42,43].

Under basal conditions, DT alone induced modest, gene-specific changes in selected EMT-associated markers, including partial modulation of CDH1, VIM, MMP2, and HIF1A. However, this response did not reproduce the full EMT-induced transcriptional profile, as MMP9 and IL-6 were reduced rather than increased, and DT alone did not enhance migratory behavior in the scratch-wound assay. Therefore, the baseline response to DT should not be interpreted as EMT induction, but rather as gene-specific responses of EMT-related signaling in already plastic breast cancer cells. The molecular effects of DT were further supported by functional assays. Both wound-healing and real-time cell analysis showed reduced migratory capacity in DT-treated cells compared with the EMT-induced group. Notably, the apparent discrepancy between the scratch-wound assay and real-time impedance analysis in non-induced MCF-7 cells may reflect differences in detection sensitivity and experimental time scale. While the wound-healing assay captures relatively early and endpoint changes in cell movement, the impedance-based method enables continuous monitoring of migration dynamics, allowing detection of delayed inhibitory effects of DT that may not be evident within the shorter observation window. Additionally, because the impedance-based migration assay is performed under serum-free conditions in the upper chamber and specifically detects cells traversing the porous membrane toward a chemoattractant, it minimizes the contribution of proliferation-related effects compared with conventional wound-healing assays. This provides complementary support that the observed differences predominantly reflect altered migratory behavior. These functional observations support the transcriptional findings, suggesting that the observed molecular changes translate into a biologically relevant attenuation of EMT-associated migratory behavior.

A significant reduction in *IL-6* expression was also observed following DT treatment. Given that IL-6 is a central mediator of inflammation-driven EMT and promotes tumor progression through STAT3 activation, its downregulation suggests suppression of pro-tumorigenic signaling [44]. Moreover, IL-6 is a key component of tumor-stroma communication, further supporting DT’s potential to interfere with microenvironmental signaling.

Together with previous reports demonstrating that DT reduces invasive behavior by modulating MMP-2/9 expression and PI3K/Akt- or MAPK-associated signaling in various tumor models, including hepatocellular carcinoma, these findings suggest that DT may attenuate selected EMT-associated molecular and functional features [45,46,47]. These observations support the interpretation that DT influences EMT-associated migratory behavior and extracellular matrix remodeling-related responses without fully reversing EMT-associated alterations.

Although direct pathway-level analyses were not performed in this study, several of the transcriptional and functional responses observed following DT treatment are consistent with biological effects previously associated with modulation of PI3K/Akt/mTOR, MAPK/ERK, and TGF-β-related signaling pathways [5,6,7,8,9,10]. In breast cancer and other malignancies, the inhibition of Akt phosphorylation directly prevents the downstream activation of major EMT-inducing transcription factors. This established molecular framework explains the dual transcriptional response captured in our MCF-7 model: the partial rescue of *CDH1* transcript levels and the simultaneous suppression of *VIM*, *MMP2*, and *MMP9* expression. Furthermore, because EMT-associated plasticity and stromal activation are strongly influenced by TGF-β-related signaling [19,20,47], the attenuation of *ACTA2*, *HGF*, and *IL-6* expression observed in our TGF-β-stimulated BJ fibroblast model suggests that diosmetin may modulate selected stromal-associated responses linked to fibroblast activation. Thus, our findings are consistent with previously reported effects of diosmetin on EMT- and TGF-β-related signaling pathways. However, direct pathway-level validation, including phosphorylation analysis of PI3K/Akt, MAPK, or TGF-β-associated mediators, was beyond the scope of the present study and should be addressed in future investigations.

The complex interplay between cancer cells and the surrounding TME plays a significant role in tumor progression and metastasis. A critical factor influencing this relationship is the expression of *HIF-1A*. Under conditions of hypoxia—a common feature of solid tumors—HIF-1α accumulates and acts as a master transcription factor, promoting a shift in cellular metabolism towards glycolysis (the Warburg effect) and stimulating the expression of genes involved in angiogenesis, cell survival, and invasion. This enhanced HIF-1A expression thus significantly contributes to the aggressive phenotype and therapeutic resistance of cancer cells [48]. Its downregulation suggests that DT may interfere with hypoxia-driven tumor progression mechanisms, potentially limiting the adaptive capacity of cancer cells under stress conditions.

Among the various components of the TME, CAFs play an important role in shaping it. CAFs facilitate tumor progression, invasion, and metastasis by secreting growth factors and pro-inflammatory cytokines [49,50]. Consequently, CAFs might serve as a promising and effective target for anti-cancer therapy. Moreover, in fibroblasts, TGF-β stimulation induced a CAF-like phenotype characterized by increased expression of *ACTA2*, *HGF*, *IL-6*, and *MMPs*, consistent with previously reported activation profiles [51,52,53].

DT treatment significantly reduced expression of these markers, indicating attenuation of fibroblast activation. Notably, decreased *ACTA2* suggests reduced myofibroblast differentiation, while downregulation of *HGF* and IL-6 indicates diminished paracrine signaling capacity.

These findings show that active stromal fibroblasts can be deactivated or normalized and suggest that natural substances such as DT could be used to remodel stromal fibroblasts associated with breast cancer and reduce their cancer-promoting effects. Thus, DT has the potential to be an effective treatment strategy targeting fibroblasts.

Additionally, we examined HA turnover genes as a readout focused on the ECM from the tumor stroma, and HA remodeling has been linked to CAF activation, tumor cell motility, and metastasis. Importantly, HA is not only a structural component but also a signaling scaffold that modulates the availability of growth factors, thereby enhancing the pro-tumorigenic environment. HA activates CAFs in both pre-malignant and malignant stroma, promoting the invasion by enhancing the mobility of both CAFs and tumor cells [54]. Elevated *HAS2* expression and *HYAL1* activity have been shown to enhance cellular motility, promote EMT, and facilitate metastasis in breast cancer models and patient tumors [55]. The effects of DT on HA metabolism primarily involve *HYAL* downregulation, which may limit extracellular matrix degradation. Given that HA turnover plays a key role in regulating cell migration and tumor progression [56], its modulation may contribute to the reduced migratory phenotype observed in this study. In combination with decreased *MMP* expression, these findings suggest a broader effect of DT on extracellular matrix remodeling.

Interestingly, under basal unstimulated conditions, DT alone induced modest upregulation of selected extracellular matrix-remodeling genes in BJ fibroblasts, particularly *MMP2* and *MMP9*. Importantly, this response was not accompanied by increased *ACTA2* expression, the key marker of CAF-like activation, suggesting that DT alone does not promote pathological fibroblast transformation. Instead, these observations may reflect adaptive or stress-associated responses of normal stromal cells. Such basal matrix-remodeling activity is a recognized feature of resting fibroblasts involved in extracellular matrix turnover and tissue maintenance; however, the precise biological significance of the transcriptional alterations observed following DT treatment requires further investigation [57,58]. In contrast, under TGF-β-stimulated conditions, DT consistently attenuated the markedly elevated expression of CAF-associated and extracellular matrix-remodeling markers. Together, these findings indicate that DT induces heterogeneous and condition-dependent transcriptional responses rather than uniformly suppressive effects across all analyzed pathways.

DT also reduced SA-β-gal activity, indicating attenuation of selected senescence-associated features in normal BJ fibroblasts. Although therapy-induced senescence may exert tumor-suppressive effects in damaged cancer cells, persistent stromal senescence induced by chemotherapeutic agents such as DOX may simultaneously contribute to chronic inflammatory signaling and tumor-supportive microenvironmental remodeling through SASP-associated activity [53,54,55]. Therefore, DT’s ability to modulate DOX-induced senescence-associated responses in BJ fibroblasts may suggest a potential stromal-modulatory effect during chemotherapy-induced stress conditions.

In this study, DOX-induced senescence in BJ cells was associated with a high proportion of SA-β-gal-positive cells, suggesting activation of a senescence-associated program. Although SASP components were not directly assessed in senescent fibroblasts, modulation of senescence-associated features by DT may be relevant in the context of stromal activation, as SASP-related signaling functionally overlaps with inflammatory and extracellular matrix remodeling pathways linked to fibroblast activation and tumor progression.

Taken together, the results of this study demonstrate that DT exerts coordinated effects on multiple components of tumor progression. By attenuating EMT-associated gene expression, reducing migratory capacity, suppressing CAF-like activation, modulating extracellular matrix remodeling, and limiting senescence-associated signaling, DT appears to modulate key processes involved in remodeling of the tumor and stromal compartments.

Importantly, the present study extends previous reports that focused primarily on the cytotoxic or chemosensitizing properties of diosmetin by demonstrating its broader modulatory effects on EMT-associated plasticity, fibroblast activation, extracellular matrix remodeling pathways, and senescence-related stromal responses, as demonstrated in parallel in vitro breast cancer models. Although the study does not directly model tumor-stroma crosstalk, it provides an integrated functional framework linking tumor-associated and stromal-associated processes relevant to breast cancer progression.

A limitation of the current study is the absence of direct co-culture systems or conditioned media experiments; however, our parallel evaluation of breast cancer cells and activated fibroblasts provides a functional foundation for future investigation of multicellular TME dynamics. In addition, EMT validation was primarily based on transcript-level analysis, supported by E-cadherin protein assessment, morphological observations, and complementary migration assays, whereas reliable detection of vimentin protein was not consistently achieved. Moreover, although migration assays demonstrated attenuation of EMT-associated motility following diosmetin treatment, direct invasion assays were not performed and should be addressed in future studies.

## 4. Materials and Methods

### 4.1. Cell Culture and Treatment

The research was performed on the breast cancer line (MCF-7) and human normal fibroblast cells (BJ) obtained from the American Type Culture Collection (ATCC, Manassas, VA, USA). The cells were maintained in Eagle’s minimum essential medium (EMEM) (Corning Inc., Corning, NY, USA) supplemented with 10% fetal bovine serum (Corning Inc., Corning, NY, USA), 100 U/mL penicillin, and 100 μg/mL streptomycin (Sigma-Aldrich, St. Louis, MO, USA). Cultures were incubated at 37 °C in a humidified atmosphere of 95% air with 5% CO_2_ in air atmosphere. The cells were detached with 0.05% trypsin EDTA (Life Technologies, Carlsbad, CA, USA).

To investigate EMT-associated cellular reprogramming, MCF-7 cells were treated for 48 h with StemXVivo EMT Inducing Media Supplement (EMTi) (R&D Systems, Minneapolis, MN, USA) according to the manufacturer’s recommended conditions to generate a mesenchymal-like variant (MCF-7-M). The StemXVivo EMTi is a commercially available defined EMT induction formulation containing recombinant human Wnt-5a and TGF-β1 together with neutralizing antibodies targeting E-cadherin, sFRP-1, and Dkk-1. The supplement is designed to promote EMT-associated signaling and phenotypic plasticity in epithelial cells. CAFs-like BJ cells were generated by TGF-β stimulation under the conditions described in the fibroblast activation protocol (Human TGF-beta 1 Recombinant Protein, Gibco, Grand Island, NY, USA). TGF-β1 inductor was added to the BJ cell culture at a final concentration of 10 ng/mL. To assess the effect of DT, cells were treated with 40 μM DT (Sigma-Aldrich, St. Louis, MO, USA). The flavonoid concentration was selected based on previous preliminary studies [13].

### 4.2. Scratch-Wound Healing Assay

To evaluate migratory capacity, MCF-7 breast cancer cells were seeded into 6-well plates (approximately 1.0 × 10^6^ cells per well) and cultured to confluence of over 70% at 37 °C in a humidified atmosphere of 95% and 5% CO_2_. The medium was then replaced with specific treatment solutions to establish four experimental cultures: MCF-7 cells treated with an equivalent concentration of DMSO (vehicle control) (MCF-7), cells treated with 40 µM DT alone (MCF-7+DT40), cells stimulated with 1 µM EMTi (MCF-7-M), and cells co-treated with 1 µM EMTi and 40 µM DT (MCF-7-M+DT40). A linear wound was mechanically created in each confluent monolayer using a sterile 1 mL micropipette tip. Following the scratch, the cells were rinsed three times with PBS to ensure the complete removal of cellular debris. The wound healing process was monitored over a 48 h incubation period. The micrographs of the scratch areas were captured at 0, 24, and 48 h post-treatment using a Nikon Eclipse Ti phase-contrast microscope (Nikon, Tokyo, Japan) at ×40 magnification equipped with NIS-Elements Imaging software version 3.22. The experiment was performed in three independent biological replicates.

### 4.3. Impedance-Based Real-Time Cell Migration Assay

Cell migration was evaluated using the xCELLigence RTCA DP system (ACEA Biosciences, San Diego, CA, USA), which enables real-time, label-free monitoring of cell migration via electrical impedance measurements. The assay was performed using CIM-Plate 16 inserts consisting of an upper and lower chamber separated by an 8 µm microporous membrane, with gold microelectrodes integrated on the underside. Before cell seeding, 160 µL of complete culture medium containing 10% FBS was added to the lower chamber of each well to serve as a chemoattractant. The assembled plate was placed in the RTCA DP station for background measurement and equilibration. MCF-7 cells were divided into four experimental cultures: control (MCF-7), MCF-7 treated with DT (MCF-7+DT40; 40 µM), EMT-induced cells (MCF-7-M), and EMT-induced cells treated with DT (MCF-7-M+DT40; 40 µM). Subsequently, 4–6 × 10^4^ cells suspended in 100 µL of serum-free medium were seeded into the upper chamber of each insert. DT (40 µM) was added to the appropriate cultures at the time of cell seeding. Impedance measurements were recorded automatically every 15 min for 96 h at 37 °C in a humidified atmosphere containing 5% CO_2_. Migration was quantified based on changes in Cell Index (CI). For data presentation, the Delta Cell Index (ΔCI) was calculated relative to the baseline time point, thereby normalizing migration curves across experimental conditions. ΔCI values correlate with the number of cells that migrated through the membrane and adhered to the electrode surface, with higher values indicating increased migratory capacity. The migration rate was further assessed by calculating the slope of the ΔCI curve during the linear phase of migration.

### 4.4. Apoptosis Analysis

Apoptosis was assessed using image cytometry with the NC-3000™ system (ChemoMetec, Allerød, Denmark) following Annexin V staining. MCF-7 cells were seeded into 6-well plates and treated for 48 h with 40 µM diosmetin (DT) under the same experimental conditions used throughout the study. Untreated cells served as the control group. After incubation, both adherent and floating cells were collected, washed with PBS, and stained using Annexin V according to the manufacturer’s protocol. Samples were analyzed using the NC-3000™ image cytometer (ChemoMetec, Allerød, Denmark). The percentages of viable, early apoptotic, late apoptotic, and necrotic cells were determined using NucleoView software version 2.1.25.12. The experiment was performed in three independent biological replicates, each containing technical triplicates.

### 4.5. SA-β-Gal Staining for Senescence Detection

SA-β-gal activity was evaluated using the Senescence β-Galactosidase Staining Kit (Cell Signaling Technology, Danvers, MA, USA) following the manufacturer’s instructions. This assay relies on the detection of β-galactosidase activity at pH 6.0, which is selectively elevated in senescent cells. BJ cells were seeded into 25 cm^3^ flasks at a density of 4 × 10^4^ cells/mL and pre-treated with 0.5 μM DOX for 48 h to induce senescence. DOX promotes senescence in various in vitro models; thus, it is considered a positive control in studies of cellular senescence induction [59,60]. After DOX exposure, cells were washed with PBS and incubated with 40 μM DT (DT) to assess its potential modulatory effect on DOX-induced senescence. Untreated cells served as the negative control, while DOX-treated cultures served as the positive control. At the end of treatment, cells were fixed using the kit’s Fixative Solution for 10–15 min at room temperature and subsequently incubated with the X-gal–containing β-galactosidase staining solution (pH 6.0). Staining was performed at 37 °C in a dry incubator (non-CO_2_) overnight. Senescent cells exhibiting blue cytoplasmic staining were visualized and counted under a Nikon Eclipse Ti phase-contrast microscope (100× magnification). For each sample, multiple random fields were imaged, and the percentage of SA-β-Gal-positive cells was calculated relative to the total number of cells. At least 100 cells per condition were counted in randomly selected fields. The experiment was repeated three times. The positive control (DOX-treated cells) was used to establish the maximum level of senescence induction, and the effect of DT (DT) was determined by comparing the percentage of SA-β-Gal cells in the DOX + DT-treated cells to the DOX group.

### 4.6. The Quantitative Real-Time PCR Analysis (qRT-PCR)

The cells were seeded into 25 cm^3^ flasks at a concentration of 4 × 10^4^ cells/mL, and the test compounds were added after reaching 70–80% confluence. After 48 h of incubation, 1 mL of TRIzol™ Reagent (Invitrogen, Carlsbad, CA, USA) was added to the culture dish to lyse the cells. Afterward, the lysate was centrifuged for 5 min at 12,000× *g* at 4 °C, and then the clear supernatant was processed according to the Chomczynski and Sacchi method [61]. The obtained RNA was reverse-transcribed using an NG dART RT-PCR kit (EURx, Gdansk, Poland) according to the manufacturer’s instructions. qPCR was performed using PowerUp SYBR Green Master Mix (ThermoFisher, Waltham, MA, USA) according to the manufacturer’s instructions on a 7500 Fast Real-Time PCR System (ThermoFisher, Waltham, MA, USA). Each qRT-PCR experiment was independently repeated three times using biological replicates, and all reactions were performed in technical triplicates. The reference genes were *RNA18SN5* and *BACT*. The statistical analysis was performed using RQ values (relative quantification, RQ = 2^−∆ΔCt^). The primer sequences used in the gene expression evaluation are presented in Table 1.

### 4.7. Western Blot Analysis of E-Cadherin Expression

Western blot analysis was performed as previously described in our earlier study [13] with minor modifications. Briefly, MCF-7 control cells, EMT-induced MCF-7 cells, and EMT-induced MCF-7 cells treated with 40 µM diosmetin (DT40) were cultured in 25-cm^2^ flasks and harvested after 48 h of incubation. Total protein was isolated using RIPA buffer (ThermoFisher, USA), and protein concentration was determined using the BCA assay (ThermoFisher, USA). Equal amounts of protein (10 µg) were separated using NuPAGE Bis-Tris gels (Invitrogen, USA) under reducing conditions and transferred onto nitrocellulose membranes. Membranes were incubated with primary mouse antibodies against E-cadherin (sc-71009, Santa Cruz Biotechnology, Dallas, TX, USA) and β-actin (sc-69879, Santa Cruz Biotechnology, Dallas, TX, USA) as a loading control, followed by alkaline phosphatase-conjugated secondary antibodies. Protein bands were visualized using the Western Breeze Chromogenic Detection Kit (Invitrogen, Carlsbad, CA, USA).

### 4.8. Cell Proliferation Assay (BrdU Incorporation)

DNA synthesis was quantified by measuring BrdU incorporation using the BrdU Cell Proliferation ELISA Kit (Abcam, Cambridge, UK) according to the manufacturer’s instructions. Briefly, BJ human fibroblast cells were seeded into 96-well plates at a density of 8 × 10^4^ cells/mL and allowed to adhere. The cells were then assigned to the following experimental groups: control fibroblasts-BJ cells treated with an equivalent concentration of DMSO, vehicle control (BJ), fibroblasts treated with 40 µM DT (BJ+DT40), senescent fibroblasts (BJ-S), and senescent fibroblasts treated with 40 µM DT (BJ-S+DT40). To facilitate incorporation into newly synthesized DNA, the BrdU labeling reagent (1x) was added to the culture medium 48 h before the endpoint measurement. At the end of the incubation period, the culture media were removed, and the cells were fixed using the provided fixative solution. Subsequently, the cells were incubated with a primary anti-BrdU antibody for 1 h at room temperature, then washed with the supplied buffer. A horseradish peroxidase (HRP)-conjugated secondary antibody was then applied for 30 min at room temperature. Signal development was achieved using a TMB substrate, and absorbance was measured at 450 nm with a PowerWave™ microplate spectrophotometer (Bio-Tek Instruments, Winooski, VT, USA). Each experiment was independently repeated three times using biological replicates, and all experimental conditions were analyzed in technical triplicates.

### 4.9. Statistical Analysis

A statistical comparison of values was performed by one-way analysis of variance (ANOVA) and post hoc multiple comparisons based on Tukey’s honest significant difference test (Tukey’s HSD test) using STATISTICA 13 software (StatSoft, Krakow, Poland). All data were expressed as mean ± SD. Statistical significance was considered to be *p* < 0.05.

## 5. Conclusions

In conclusion, our study demonstrates that DT modulated several tumor- and stromal-associated processes relevant to breast cancer progression. In MCF-7 cells, DT attenuated EMT-induced migration, was associated with partial restoration of epithelial-associated features, and reduced the expression of several pro-inflammatory and extracellular matrix remodeling-related genes, including MMP2, MMP9, IL6, and HIF1A. In BJ fibroblasts, DT attenuated TGF-β-induced CAF-like activation, as reflected by reduced expression of ACTA2, HGF, IL6, MMP2, and MMP9. Beyond its effects on EMT-associated plasticity and stromal activation, DT also modulated hyaluronan turnover-related transcripts and attenuated selected senescence-associated features in DOX-treated fibroblasts. Collectively, these findings indicate that DT influences multiple processes associated with tumor cell plasticity and stromal activation in parallel in vitro models. While additional mechanistic, multicellular, and in vivo studies are required, DT may warrant further investigation as a potential adjunctive compound in combination-based approaches for breast cancer.

## Figures and Tables

**Table 1:** Primers used to assess gene expression.

Target	Gene Name	Forward Sequence	Reverse Sequence
*ACTA2*	Actin Alpha 2, Smooth Muscle	CTATGCCTCTGGACGCACAACT	CAGATCCAGACGCATGATGGCA
*HGF*	Hepatocyte Growth Factor	GAGAGTTGGGTTCTTACTGCACG	CTCATCTCCTCTTCCGTGGACA
*CDH1*	E-cadherin	TTTGAAGATTGCACCGGTCG	AGAAACGGAGGCCTGATGG
*VIM*	Vimentin	GAGTCCACTGAGTACCGGAG	ACGAGCCATTTCCTCCTTCA
*MMP2*	Matrix Metallopeptidase 2	GCGTCTTCCCCTTCACTTTC	ATAGGGTACATGAGCGCCTC
*MMP9*	Matrix Metallopeptidase 9	CAGCCCTGCAAGTTTCCATT	GTTGCCCAGGAAAGTGAAGG
*IL-6*	Interleukin-6	AGACAGCCACTCACCTCTTCAG	TTCTGCCAGTGCCTCTTTGCTG
*HIF-1A*	HIF-1 alpha	TATGAGCCAGAAGAACTTTTAGGC	CACCTCTTTTGGCAAGCATCCTG
*HAS1*	Hyaluronan synthase 1	CTGCGATACTGGGTAGCCTTCA	CCAGGAACTTCTGGTTGTACCAG
*HYAL1*	Hyaluronidase 1	GACACGACAAACCACTTTCTGCC	ATTTTCCCAGCTCACCCAGAGC
*RNA18SN5*	18S ribosomal RNA	ACCCGTTGAACCCCATTCGTGA	GCCTCACTAAACCATCCAATCGG
*BACT*	Actin, beta	CACCATTGGCAATGAGCGGTTC	AGGTCTTTGCGGATGTCCACGT

## Data Availability

The original contributions presented in this study are included in the article/Supplementary Materials. Further inquiries can be directed to the corresponding author.

## Supplementary Materials

The following supporting information can be downloaded at: https://www.mdpi.com/article/10.3390/molecules31122111/s1. Table S1: Quantitative numerical data representing gene expression changes in MCF-7 and MCF-7-M breast cancer cell lines treated with the DT40; Table S2: Percentage of relative wound area in the scratch-wound assay in MCF-7 and MCF-7-M cell lines treated with DT40; Table S3: Quantitative numerical data representing gene expression changes in BJ fibroblast and CAFs-like BJ cell lines treated with the DT40; Table S4: Assessment of senescence-associated β-galactosidase activity in BJ and BJ-S lines; Table S5: Proliferation analysis of BJ and BJ-S cell lines based on BrdU incorporation; Figure S1: The original blots used for the evaluation of E-cadherin protein expression; Figure S2: Assessment of apoptosis in MCF-7 cells following treatment with 40 µM diosmetin (DT); Figure S3: Representative microscopic images acquired at ×40 magnification at 0 h, 24 h, and 48 h, illustrating relative wound area over time; Figure S4: Representative images of SA-β-Gal staining in all experimental cultures.

## Abbreviations

The following abbreviations are used in this manuscript:

α-SMA	alpha-smooth muscle actin
CAFs	cancer-associated fibroblasts
DT	diosmetin
DOX	doxorubicin
EMT	epithelial–mesenchymal transition
HA	hyaluronan
HAS	hyaluronan synthase
HYAL1	hyaluronidase
HIF-1α	hypoxia-inducible factor 1 alpha
IL-6	interleukin-6
MMPs	matrix metalloproteinases
SA β-gal	senescence-associated β-galactosidase
SASP	senescence-associated secretory phenotype
TME	tumor microenvironment
