# Supplementary material for: Diosmetin Modulates EMT-Associated Plasticity and Fibroblast-Activation Markers in Parallel Breast Cancer In Vitro Models
Source: Molecules. 2026 Jun 16;31(12):2111. doi: 10.3390/molecules31122111 (PMC13304686; doi:10.3390/molecules31122111)
Supplement: Supplementary file 1 [file molecules-31-02111-s001.zip › molecules-4321241-supplementary.pdf]

Supplementary Information

Table S1. Quantitative numerical data representing gene expression changes in MCF-7 and MCF-7-M breast cancer cell lines treated with the DT40. MCF-7-M denotes the mesenchymal-like variant of MCF-7 cells with an EMT phenotype. MCF-7 + DT40 indicates parental cells treated with DT40, whereas MCF-7-M + DT40 refers to mesenchymal-like cells treated with DT40. Mean (RQ) – mean relative quantification; SD – standard deviation; N – number of replicates.

	**MCF-7**			**MCF-7+DT40**			**MCF-7-M**			**MCF7-M +DT40**		
	**Mean (RQ)**	**SD**	**N**	**Mean (RQ)**	**SD**	**N**	**Mean (RQ)**	**SD**	**N**	**Mean (RQ)**	**SD**	**N**
CDH1	1.00	0.04	3	1.96	0.04	3	0.53	0.02	3	1.58	0.03	3
VIM	1.00	0.05	3	2.19	0.15	3	8.79	0.23	3	2.76	0.09	3
MMP2	1.00	0.02	3	2.39	0.00	3	2.78	0.06	3	1.26	0.01	3
MMP9	1.00	0.03	3	0.69	0.09	3	2.83	0.11	3	0.69	0.13	3
IL6	1.00	0.02	3	0.36	0.06	3	2.18	0.10	3	0.97	0.01	3
HIF1A	1.00	0.06	3	1.20	0.05	3	1.88	0.16	3	0.93	0.03	3
HAS1	1.00	0.08	3	1.30	0.02	3	1.41	0.05	3	1.04	0.06	3
HYAL1	1.00	0.01	3	0.82	0.06	3	1.54	0.01	3	0.80	0.06	3

Table S2. Percentage of relative wound area in the scratch-wound assay in MCF-7 and MCF-7-M cell lines treated with DT40. Data are presented as mean ± SD at 0 h, 24 h, and 48 h. MCF-7-M denotes the mesenchymal-like variant of MCF-7 cells with an EMT phenotype. MCF-7 + DT40 indicates parental cells treated with DT40, whereas MCF-7-M + DT40 refers to mesenchymal-like cells treated with DT40.

Time	MCF-7	MCF-7 + DT40	MCF-7-M	MCF-7-M + DT40
0 h	57.17 ± 0.80	52.37 ± 0.55	45.10 ± 0.31	47.24 ± 0.75
24 h	46.61 ± 0.17	43.01 ± 0.92	29.32 ± 0.73	39.11 ± 0.94
48 h	40.91 ± 0.63	35.37 ± 0.77	19.21 ± 0.57	44.43 ± 0.69

Table S3. Quantitative numerical data representing gene expression changes in BJ fibroblast and CAFs-like BJ cell lines treated with the DT40. BJ represents the parental human skin fibroblast cell line. CAFs-like BJ denotes the cancer-associated fibroblast-like variant of BJ cells, characterized by an activated/EMT phenotype. BJ + DT40 indicates parental cells treated with 40 µM of DT, whereas CAFs-like BJ + DT40 refers to the mesenchymal-like (CAFs-like) variant treated with DT40. Mean (RQ) – mean relative quantification; SD – standard deviation; N – number of replicates.

	**BJ**			**BJ+DT40**			**CAFs-like BJ**			**CAFs-like-BJ+DT40**		
	**Mean (RQ)**	**SD**	**N**	**Mean (RQ)**	**SD**	**N**	**Mean (RQ)**	**SD**	**N**	**Mean (RQ)**	**SD**	**N**
ACTA2	1.00	0.08	3	0.33	0.04	3	1.44	0.25	3	0.65	0.04	3
HGF	0.94	0.31	3	1.72	0.16	3	4.22	0.46	3	0.55	0.01	3
MMP2	1.00	0.03	3	2.14	0.05	3	4.32	0.41	3	2.76	0.16	3
MMP9	1.00	0.03	3	3.50	0.16	3	8.32	0.23	3	3.58	0.37	3
IL-6	1.01	0.17	3	1.36	0.10	3	6.29	0.13	3	1.30	0.04	3
HIF1A	1.00	0.03	3	0.20	0.01	3	1.10	0.02	3	0.56	0.04	3
HAS1	1.01	0.16	3	1.07	0.20	3	0.24	0.01	3	0.18	0.04	3
HYAL1	1.00	0.02	3	0.12	0.02	3	1.57	0.15	3	0.36	0.01	3

Table S4. Assessment of senescence-associated β-galactosidase activity in BJ and BJ-S lines. The table presents the percentage of cells showing positive staining for the senescence marker following DT40 treatment. The experimental groups were defined as follows BJ – untreated cells (control); BJ+DT40 – fibroblasts treated with 40 μM diosmetin; BJ-S – senescent fibroblasts; BJ-S+DT40 – senescent fibroblasts treated with 40 μM DT40. Data are presented as mean (Mean) ± standard deviation (SD) for three independent experiments (N=3).

	BJ			BJ+DT40			BJ-S			BJ-S+DT40		
	**Mean**	**SD**	**N**	**Mean**	**SD**	**N**	**Mean**	**SD**	**N**	**Mean**	**SD**	**N**
**β-galactosidase + (% cells)**	12.07	3.52	3	16.20	3.95	3	80.30	7.60	3	48.83	5.42	3

Table S5. Proliferation analysis of BJ and BJ-S cell lines based on BrdU incorporation. Data represent the percentage of BrdU-positive cells (exhibiting active DNA synthesis) in control (BJ) and DT40-treated groups. The experimental groups were defined as follows: BJ – untreated cells (control); BJ+DT40 – cells treated with 40 μM DT40; BJ-S – cells treated with 1 μM DOX (senescent BJ cells); BJ-S+DT40 – DOX-induced senescent BJ cells treated with 40 μM DT40. Results are expressed as mean (Mean) ± standard deviation (SD) for three independent experiments (N=3).

	BJ			BJ+DT40			BJ-S			BJ-S+DT40		
	**Mean**	**SD**	**N**	**Mean**	**SD**	**N**	**Mean**	**SD**	**N**	**Mean**	**SD**	**N**
**% of control**	100	3	3	100	7	3	14.33	3.06	3	34.67	7.51	3

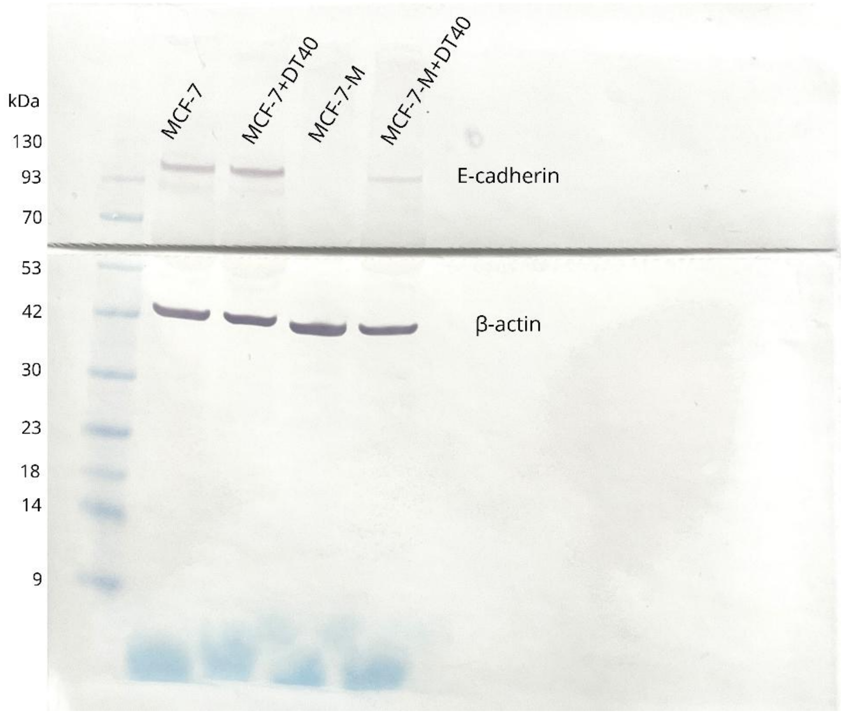

Figure S1. The original blots used for the evaluation of E-cadherin protein expression.

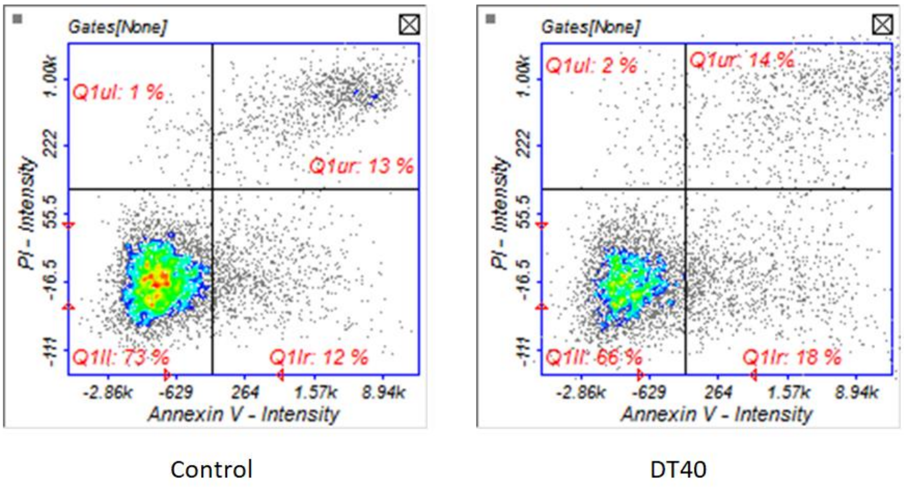

Figure S2. Assessment of apoptosis in MCF-7 cells following treatment with 40 μM diosmetin (DT). Representative Annexin V-based image cytometry histograms obtained using the NC-3000™ system (ChemoMetec, Denmark) after 48 h incubation with DT. Untreated cells served as the control group. The analysis demonstrated no marked increase in apoptotic cell death in DT-treated cells compared with control cultures under the experimental conditions used in the present study.

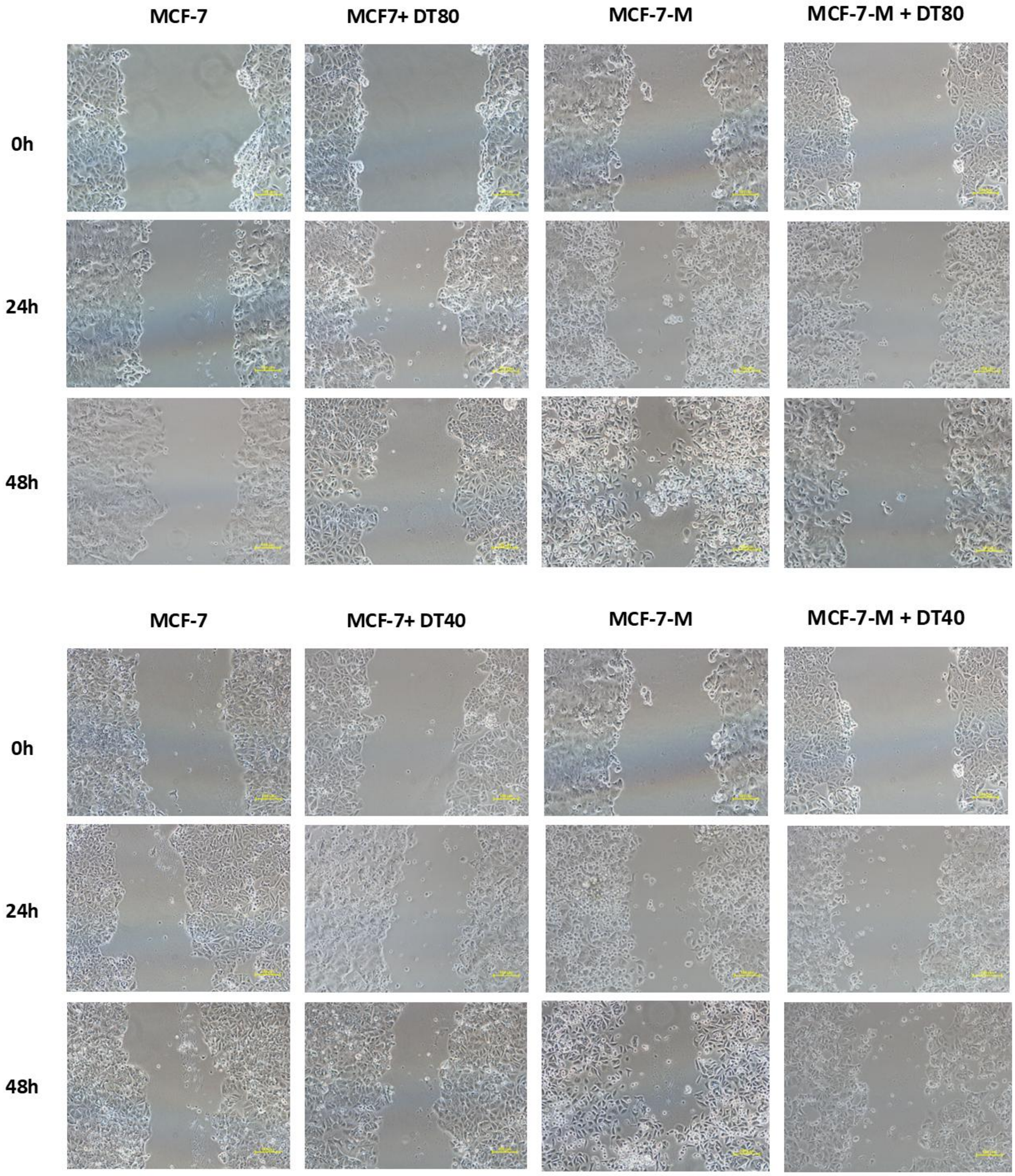

Figure S3. Representative microscopic images acquired at ×40 magnification at 0 h, 24 h, and 48 h, illustrating relative wound area over time. Representative images from independent experiments are shown. Scale bars = 100 μm

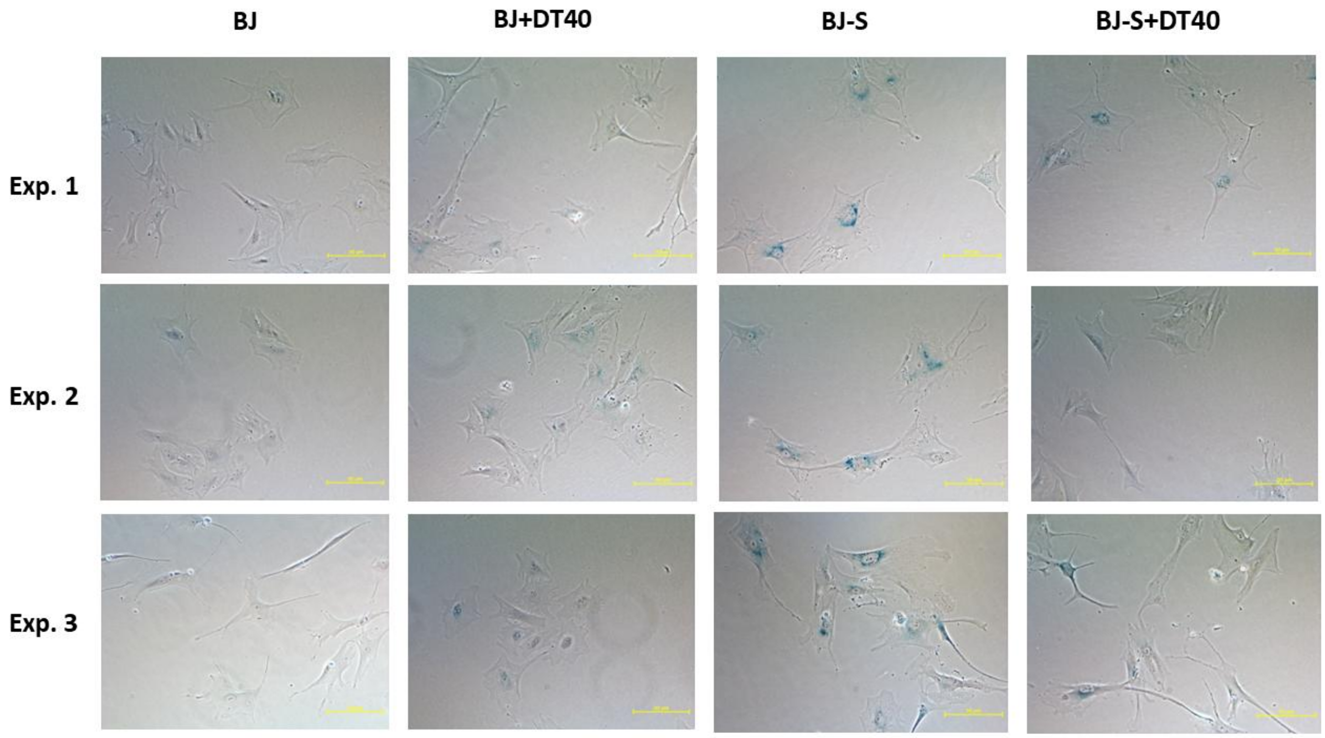

Figure S4. Representative images of SA-β-Gal staining in all experimental cultures.
Cells were stained for senescence-associated β-galactosidase (SA-β-Gal). Representative images from three independent experiments are shown for each condition. Images were acquired from randomly selected fields. Scale bars = 50 μm
